# A Label-Free Immunosensor Based on Gold Nanoparticles/Thionine for Sensitive Detection of PAT Protein in Genetically Modified Crops

**DOI:** 10.3389/fchem.2021.770584

**Published:** 2021-12-07

**Authors:** Qianwen Yang, Yu Wang, Xiaofeng Liu, Hua Liu, Huifang Bao, Jinbin Wang, Haijuan Zeng

**Affiliations:** ^1^ Key Laboratory of Agricultural Genetics and Breeding, The Biotechnology Research Institute, Shanghai Academy of Agricultural Sciences, Shanghai, China; ^2^ School of Life Science and Engineering, Lanzhou University of Technology, Lanzhou, China; ^3^ Crops Ecological Environment Security Inspection and Supervision Center (Shanghai), Ministry of Agriculture and Rural Affairs, Shanghai, China; ^4^ Institute of Applied Microbiology, Xinjiang Academy of Agricultural Sciences, Urumchi, China

**Keywords:** immunosensor, thionine, gold nanoparticles, label free, genetically modified crops

## Abstract

Genetically modified (GM) crops containing phosphinothricin acetyltransferase (PAT) protein has been widely planted worldwide. The development of a rapid method for detecting PAT protein is of great importance to food supervision. In this study, a simple label-free electrochemical immunosensor for the ultrasensitive detection of PAT protein was constructed using thionine (Thi)/gold nanoparticles (AuNPs) as signal amplification molecules and electrochemically active substances. Under optimum conditions, the limits of detection of the sensor for soybean A2704-12 and maize BT-176 were 0.02% and 0.03%, respectively. The sensor could detect crops containing PAT protein and had no cross-reaction with other proteins. After storage at 4°C for 33 days, the sensor still retained 82.5% of the original signal, with a relative standard deviation (RSD) of 0.92%. The recoveries of the sensor for soybean A2704-12 and maize BT-176 were 85%–108% and 98%–113%, respectively. The developed PAT-target immunosensor with high sensitivity, specificity, and satisfactory reproducibility and accuracy will be a useful tool in the trace screening of GM crops. Moreover, this design concept can be extended to other proteins by simply changing the antibody.

## 1 Introduction

In recent years, the cultivation area of genetically modified (GM) crops has rapidly expanded and reached 1.904 billion hectares (James, 2019). Herbicide-resistant crops are one of the most important and widest-planted GM crops (Mathur et al., 2017) and include soybean, maize, cotton, and rapeseed. Phosphinothricin acetyltransferase (PAT) protein is encoded by the *bar* or the *pat* gene and makes crops resistant to the herbicide glyphosate (Hérouet et al., 2005). In China, GM crops and products are strictly subject to mandatory labeling by GM organic management; however, unauthorized GM crops or products still appear in the field and markets from time to time (Yue et al., 2019). Thus, it is necessary to find an effective and rapid method to strengthen the detection and supervision of GM crops and products.

At present, increasingly precise instruments and methods are being applied to detect GM crops, including polymerase chain reaction (PCR) assays and real-time immune-PCR (IPCR) (Liu et al., 2016; Geoffrey et al., 2018). As a DNA-based method, PCR can qualitatively and quantitatively analyze ingredients in GM crops. However, PCR methods depend on thermal cycler, which are complicated and time consuming, and fail to detect exogenous proteins (Grel et al., 2011). Methods based on protein-specific expression, such as enzyme-linked immunosorbent assay (ELISA), the test strip, and Western blot (Wang et al., 2015; Albright et al., 2016; Zeng et al., 2020), have also been applied to GM crops. While these methods are fast, they fail to sensitively and quantitatively detect the protein in GM crops. In addition, some new effective and rapid detection methods, including surface plasmon resonance, loop-mediated isothermal amplification (LAMP), and electrochemical immunosensors, are also being used for GM crop detection (Ming et al., 2015; Shen et al., 2016; Lu et al., 2018).

To construct a sensitive, selective, and stable immunosensor, many strategies have been used. Nanomaterials with a large surface area and good conductivity have been utilized as a substrate and to improve electron transfer capability (Lv et al., 2018). Functional molecules with active groups have been used to increase the effective binding sites. To date, various nanomaterials have been used to enhance the detection signal of immunosensor, such as gold nanoparticles (Choosang et al., 2021), quantum and carbon dots (Yang et al., 2020; Qin et al., 2019), and metal oxide nanomaterials (Dekany and Sebok and Daniel, 2015). Thionine (Thi) is a cationic phenothiazine dye that is usually used as an electrochemical indicator. As a functional monomer with two amino groups, it has exceptional electrochemical stability with good electron transfer ability (García-Mendiola et al., 2020). Because of these properties, Thi is widely used in the field of electrochemistry (Fan et al., 2019; Wang et al., 2020).

In this study, gold nanoparticles (AuNPs) were modified onto the electrode surface by electrodeposition to increase the electron transfer. Additionally, Thi was connected to the surface *via* Au–S bonds to dramatically enhance the electrochemical signal and was also used for conjunction with the monoclonal antibody (mAb). Based on this method, a label-free electrochemical immunosensor for PAT protein was constructed with high sensitivity, specificity, and reproducibility. Furthermore, this simple concept provides a useful approach for the detection of transgenic proteins and can also be applied in other field by changing the recognition elements.

## 2 Materials and Methods

### 2.1 Materials and Apparatus

The mAb against the PAT protein was purchased from Artron (Jinan, China). Glutaraldehyde, chloroauric acid, bovine serum albumin (BSA), and Thi were purchased from Sigma (St. Louis, MO, USA). Seed powder standards of GM crops (5%, 100%, and 1%) used for the immunosensor test were obtained from ERM (Geel, Belgium), AOCS (Urbana, IL, USA), and the Ministry of Agriculture (Beijing, China), as listed in Supplementary Table S1. All chemicals and solvents were of analytical grade.

All the electrochemical measurements used a three-electrode system on a computer-controlled CHI 660E electrochemical workstation (Chenhua, Shanghai, China). A glassy carbon electrode (GCE, d = 3 mm) was used as the working electrode, and the auxiliary and reference electrodes employed a platinum and Ag/AgCl electrode, respectively. The micromorphology of the materials coated on the GCE was verified by a scanning electron microscope (SEM, Hitachi, Japan).

### 2.2 Preparation of the Phosphinothricin Acetyltransferase Electrochemical Immunosensor

**Scheme 1:**
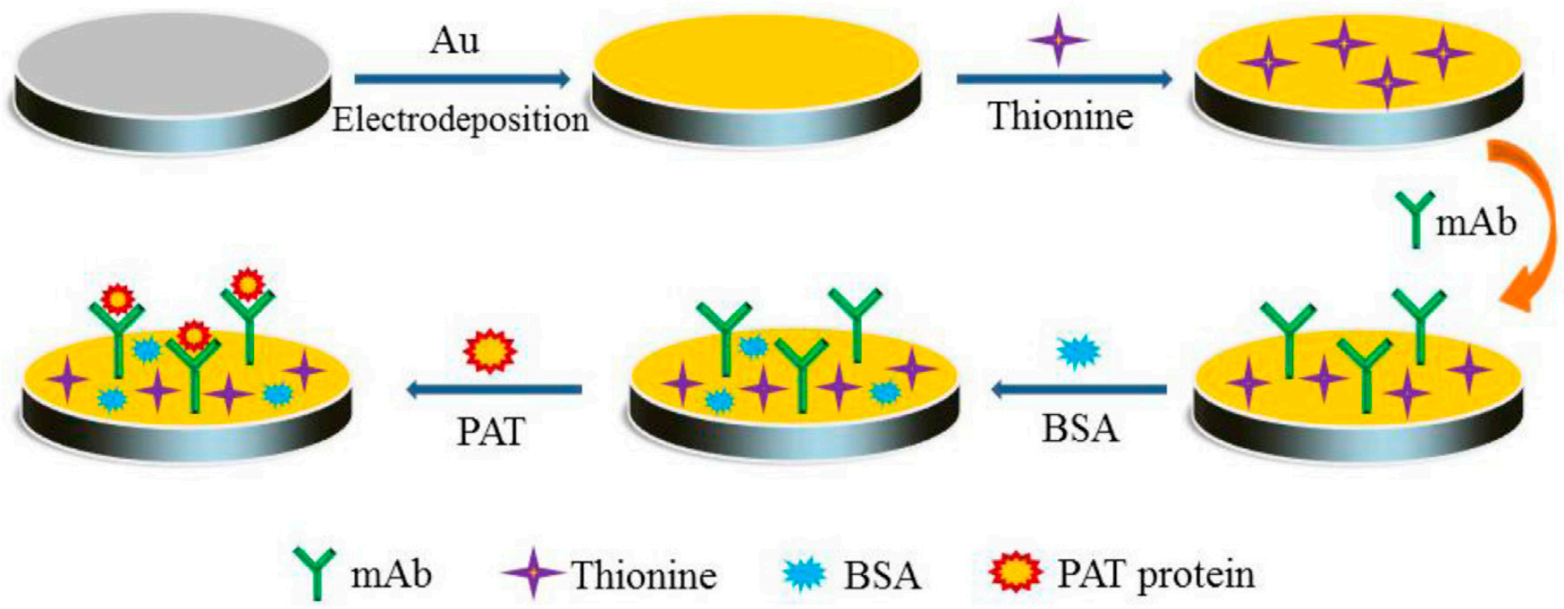
Schematic diagram of the electrochemical immunosensor based on gold nanoparticles (AuNPs)/thionine (Thi) for the sensitive and rapid detection of phosphinothricin acetyltransferase (PAT) protein in genetically modified (GM) crops.

Prior to modification, the GCE was polished with 1 μm, 0.3 µm, and 0.05 µm aluminum powder sequentially on polishing paper, with sonication in double-distilled water between each polishing step. The cleaned GCE was immersed in HAuCl_4_ solution (1%) and electrodeposited on a cyclic voltammeter (CV) with measurements from−0.2 to +0.6 V (Zhang et al., 2011). A second layer was formed by dropping Thi solution (1.0 mg/ml, 5 μl) onto the surface of GCE and storing it at 4°C overnight. Afterward, the PAT-mAb was dropped onto the GCE and incubated at 37°C for 40 min to form the third layer. Finally, 5 μl of PBS (0.01 M, pH 7.4) containing 5% BSA was dropped onto the electrode to block remaining active sites. Each modified process was followed by washing with double-distilled water. The prepared electrode was stored at 4°C for subsequent use. The process of immunosensor preparation is illustrated in Scheme 1.

### 2.3 Electrochemical Measurements

The electrochemical measurement depends on the current changes before and after the antigen–antibody reaction. When the antigen–antibody immune complex is formed, the electron transfer is hindered and the peak current response is reduced. Differential pulse voltammetry (DPV) measurements were recorded from 0 to +0.4 V at a scan rate of 50 mV/s in 0.1 M PBS solution (pH 7.4) containing 5 mM [Fe(CN)_6_]^3−/4−^ and 0.1 M KCl to detect the PAT protein. The amplitude, pulse width, sampling width, and quiet time were 0.05 V, 0.06 s, 0.02 s, and 2 s, respectively. The assembly process of the immunosensor was characterized step-by-step using CV measurements with [Fe(CN)_6_]^3−/4−^ as the redox probe. All experimental measurements were performed at room temperature.

### 2.4 Sample Analysis

The PAT protein was extracted from different crop seed powder standards using PBS buffer (0.01 M, pH 7.4) at a mass volume ratio of 1:3. After adding the PBS into the seed powder, the mixture was shaken at room temperature for 5 min and centrifuged at 6,000 rpm for 5 min to remove the pellet. Crop supernatants were stored at 4°C for follow-up tests. Crop supernatants with different transgenic contents were diluted with corresponding blank crop supernatants. Five microliters of different samples were dropped onto the prepared working electrode incubation at 37°C for 40 min.

## 3 Results

### 3.1 Characterization of the Modified Process

**FIGURE 1:**
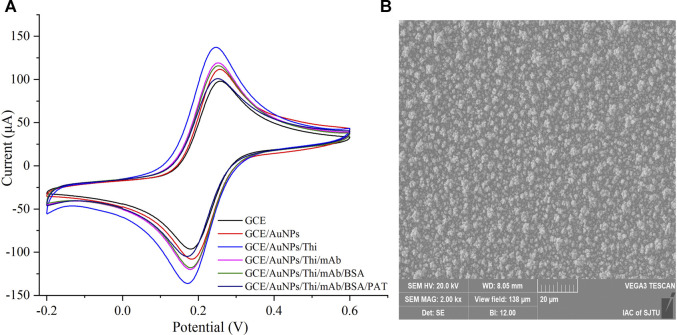
Characterization of the electrochemical immunosensor. **(A)** The stepwise assembly process of the immunosensor was detected by cyclic voltammeter measurements. **(B)** The scanning electron microscope (SEM) image of gold nanoparticles (AuNPs) deposited on the electrode surface.

The stepwise assembly process of the immunosensor was detected by CV in PBS (0.1 M, pH 7.4) containing 5 mM [Fe(CN)_6_]^3−/4−^ and 0.1 M KCl at a scan rate of 50 mV/s, as shown in Figure 1A. The bare GCE had a low-level peak current value, while the electrodeposited AuNPs could largely increase the surface area of electrode and promote electron transfer. When the Thi was bound to the electrode surface, the peak current increased significantly. As the antibody and BSA hindered the electron transport, the current gradually decreased when the antibody and BSA were coupled to the electrode. Finally, the peak current continued to decrease followed by incubation with PAT. The change in current on the electrode surface was obvious, indicating that the immunosensor had been successfully modified. Furthermore, the enhancement of the current by AuNPs/Thi was sufficient.

AuNPs, as a low-dimensional functional material, has been reported to increase the surface area of sensors and the immobilization of antibodies (Zhang et al., 2011). Modification with AuNPs promoted the electron transfer between the oxidized compound and the sensors (Lu et al., 2018). In this study, AuNPs were electrodeposited onto the surface of GCE to promote the electronic transmission and amplify the sensor signals. The surface morphology of the modified electrode was investigated using SEM. As shown in Figure 1B, AuNPs formed a uniform film on the surface, which increased the surface area and greatly improved the loading capacity of the GCE.

### 3.2 Optimization of the Thionine Conditions

**FIGURE 2:**
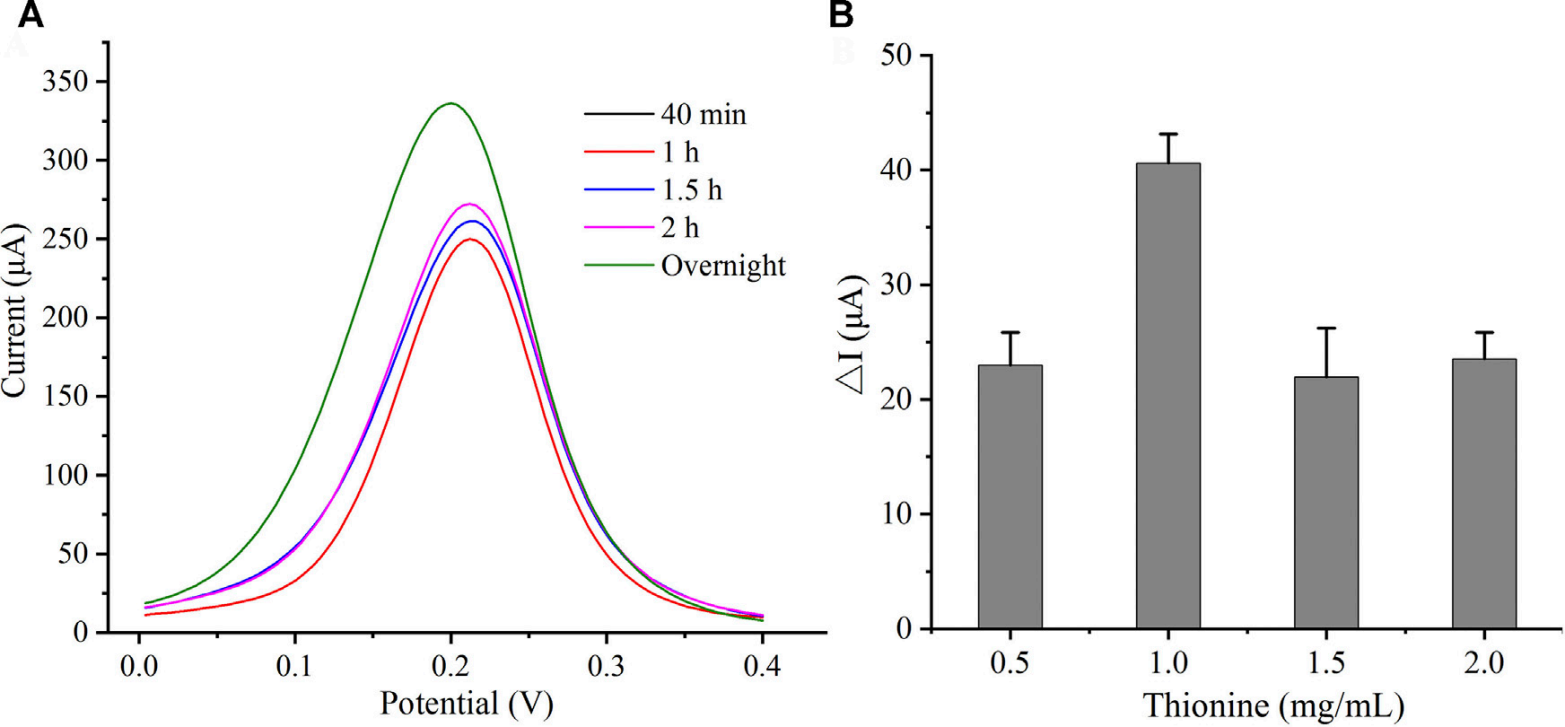
Condition optimization of Thi. **(A)** Optimization of time. **(B)** Optimization of Thi concentration.

To obtain the best sensor response, the concentration and incubation time of Thi were optimized. As displayed in Figure 2A, with the increase in incubation time, the current signal gradually enhanced, and 4°C incubation overnight resulted in a maximum current response. Since there were no significant differences between 40 min and 1 h, the data overlapped in the figure. The influence of Thi concentration on current response is presented in Figure 2B. The peak current increased with the increase in Thi concentration. When the Thi concentration was higher than 1.0 mg/ml, the peak current decreased. This may be due to excessive Thi accumulation hindering the electron transfer on the electrode surface. Therefore, 1.0 mg/ml of Thi and an overnight incubation were selected as the optimal conditions for further testing.

### 3.3 Performance analysis of Phosphinothricin Acetyltransferase Immunosensor

#### 3.3.1 Sensitivity of the Immunosensor

**FIGURE 3:**
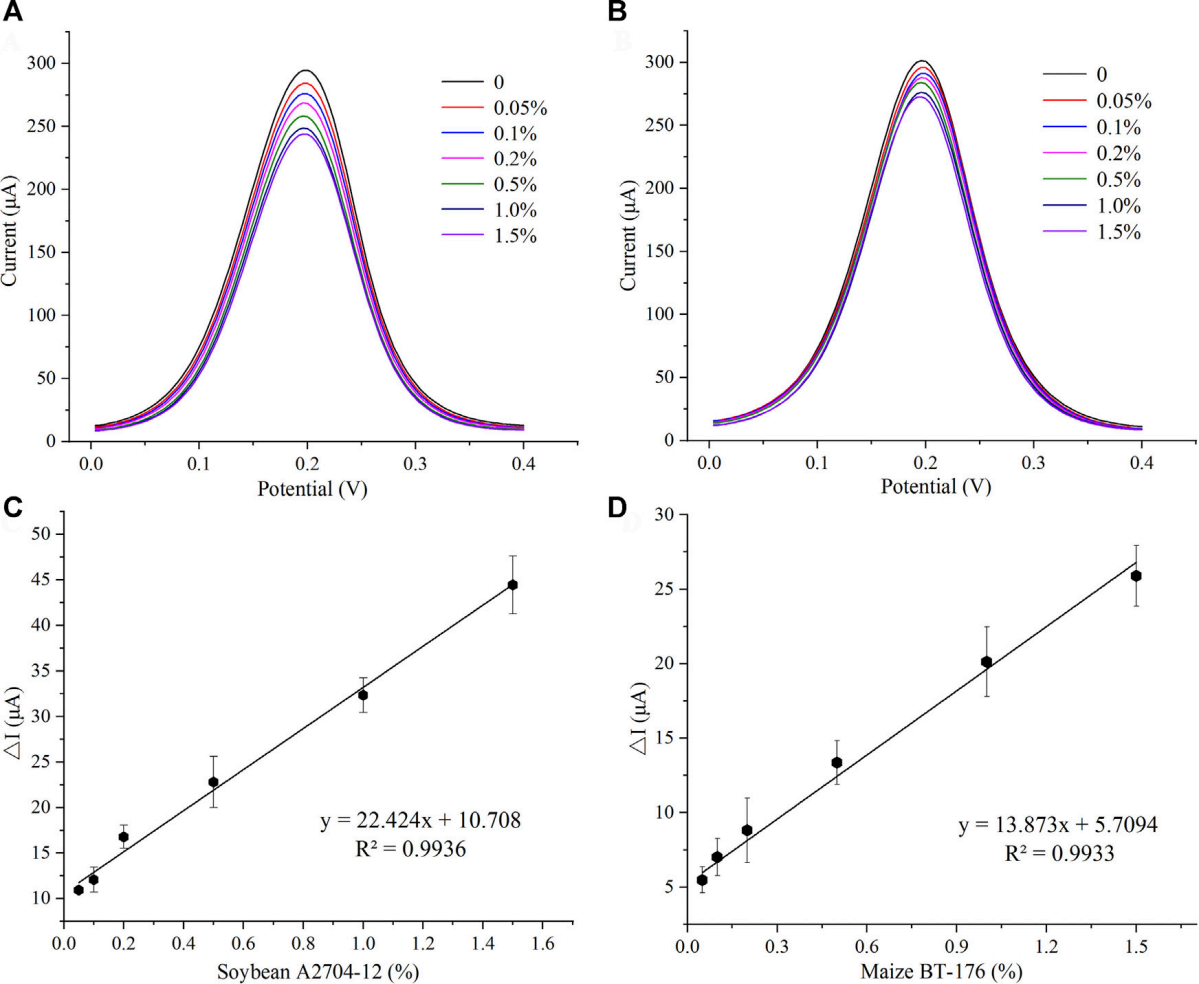
Sensitivity of the immunosensor. **(A)** Differential pulse voltammetry (DPV) peak current for different concentrations of soybean A2704-12. **(B)** DPV peak current for different concentrations of maize BT-176. **(C)** Calibration curve of the immunosensor toward soybean A2704-12 (*n* = 3). **(D)** Calibration curve of the immunosensor toward maize BT-176 (*n* = 3).

Under the optimal experimental parameters, the sensitivity of the developed PAT-targeted immunosensor was evaluated by DPV measurements. The current responses with different concentrations of soybean A2704-12 and maize BT-176 are shown in Figures 3A, B (from top to bottom, the concentrations of PAT in PBS buffer were in the range of 0.05%–1.5%). The peak current decreased as the concentration of GM crops increased, which was due to the hindrance of electron transfer with the increase of PAT protein on the electrode surface. The corresponding calibration curves for soybean A2704-12 and maize BT-176 are shown in Figures 3C, D. The linear regression equations were ΔI = 22.424x + 10.708 (*R* ^2^ = 0.9936) and ΔI = 13.873x + 5.7094 (*R* ^2^ = 0.9933), where ΔI was the current signal (μA) change and x was the PAT concentration. The limit of detection was 0.02% for A2704-12 and 0.03% for BT-176 samples (S/N = 3), which was the sensitivity of the developed immunosensor.

#### 3.3.2 Selectivity and Stability of the Immunosensor

**FIGURE 4:**
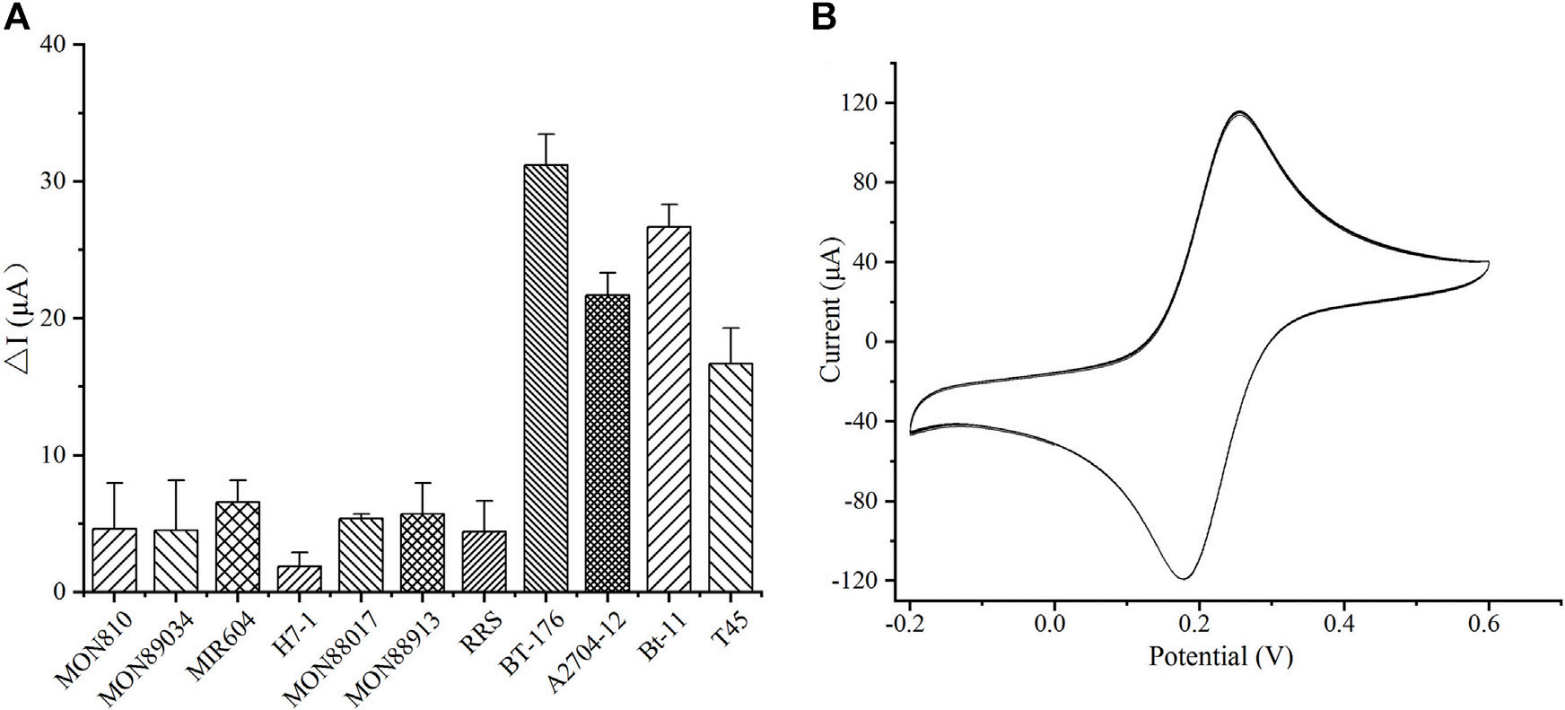
Specificity and stability of the developed immunosensor. **(A)** The specificity of the sensors. **(B)** The stability of the sensors by cyclic voltammeter (CV) measurements.

To estimate the selectivity of the immunosensor, common GM crops containing different GM proteins were tested. GM crops at contents of 5%, i.e., maize BT-176 (BT-Cry1Ac/PAT), maize MIR604 (BT-Cry3A), maize MON89034 (BT-Cry1A105/Cry2Ab), maize MON88017 (CP4-EPSPS/Cry3Bb1), soybean RRS (CP4-EPSPS), cotton MON88913 (CP4-EPSPS), and sugar beet H7-1 (CP4-EPSPS), were investigated. Additionally, the GM crops at contents of 1% including maize MON810 (BT-Cry1Ab), maize BT-11 (BT-Cry1Ab/PAT), soybean A2704-12 (PAT), and rapeseed T45 (PAT) were also examined. A blank signal was obtained from corresponding non-GM crops. As shown in Figure 4A, the peak current change in crops containing PAT protein was significantly higher than the others. The results indicated that the sensor could detect crops containing PAT protein, and the detection ability was not affected by other proteins such as CP4-EPSPS, BT-Cry1Ab, BT-Cry3A, and BT-Cry1A105/Cry2Ab.

The stability of the developed sensor was evaluated. The constructed immunosensor still maintained 82.5% of the initial current value after storage at 4°C for 33 days. The sensor was then successively scanned under continuous CV for 15 cycles after storage for 33 days (Figure 4B), and the relative standard deviation (RSD) of the response was 0.92%. Thus, the constructed immunosensor demonstrated acceptable stability and satisfactory repeatability.

#### 3.3.3 Recovery Experiment

The accuracy of the constructed immunosensor was evaluated using BT-176 and A2704-12 standards with five different concentrations (0.05%, 0.1%, 0.5%, and 1%). Each concentration had three parallel tests, and the results are shown in Table 1. The average recoveries of the maize and soybean samples were 98%–113% and 85%–108%, respectively. The RSDs were all less than 15.0%, which further confirmed that the electrochemical sensor was suitable for the detection of PAT protein and had good accuracy.

**TABLE 1:** Recoveries of different genetically modified content detected by the immunosensor (*n* = 3).

Sample	Added (%)	Detected (‾x ± SD, %)	Recovery (‾x ± RSD, %)
BT-176	0.05	0.0498 ± 0.0072	99.6 ± 14.4
	0.1	0.1075 ± 0.0055	107.5 ± 5.1
	0.5	0.5648 ± 0.0294	113.0 ± 5.2
	1	0.9844 ± 0.1024	98.4 ± 10.4
A2704-12	0.05	0.0429 ± 0.0058	85.8 ± 13.5
	0.1	0.0955 ± 0.0108	95.5 ± 11.3
	0.5	0.5392 ± 0.0766	107.8 ± 14.2
	1	0.9651 ± 0.0820	96.5 ± 8.5

## 4 Discussion

According to previous reports, the immunosensor based on AuNPs/Thi/CMWCNTs (carboxylated multiwalled carbon nanotubes) for measuring interleukin-6 (IL-6) has been developed with high sensitivity (Wang et al., 2020). To the best of our knowledge, the AuNPs/Thi-modified electrochemical immunosensor for ultrasensitive detection of PAT protein has not been reported before. A brief summary of the different methods for the detection of GM crops is presented in Table 2. Each detection method has its own significance and value for the final determination of food quality and safety (Nazir et al., 2019). PCR (Cao et al., 2018) and real-time PCR methods (Yao et al., 2018) are used for detecting GM crops at nucleic acid levels. Immunochromatographic strip (ICS) testing can rapidly detect the targets with high specificity but generally low sensitivity. The previously reported electrochemiluminescence (ECL) method showed high sensitivity (Zhang et al., 2020); however, the materials used to modify the electrode were unfriendly to the environment and human health (Li et al., 2021). The main advantages of this study were as follows: 1) AuNPs were electrodeposited on the electrode surface without complicated procedures. 2) AuNPs and Thi were connected by an Au–S bond, which benefited the long-term stability and sensitivity of sensors (Xu et al., 2015). This simple modification scheme can enhance the conductivity of electrodes and enlarge the surface area to provide more binding sites.

**TABLE 2:** Performance comparison with different detection methods of genetically modified crops.

Method	Material	Detection target	LOD	Time	Reference
LAMP	—	P35S; cp4epsps; pat; Cry1Ab/Ac	0.5%	1 h	Takabatake et al. (2018)
Real-time PCR	—	P35S and NOS	0.005%	2 h	Junichi et al. (2019)
Immunochromatographic strip	AuNPs	CP4-EPSPS	0.05%	5–10 min	Zeng et al. (2020)
Label-free ECL immunosensor	Carbon nanospheres	BT-Cry1Ab	0.02%	1 h	Gao et al. (2017)
ECL	Carbon nanoparticles	PAT/bar	0.02%	1 h	Gao et al. (2018)
Biosensor (RPA-LFS)	—	MON810	0.1%	25 min	Zhang et al. (2021)
Label-free immunosensor	AuNPs/Thi	PAT	0.02%/0.03%	40 min	This study

Note. LAMP, loop-mediated isothermal amplification; PCR, polymerase chain reaction; ECL, electrochemiluminescence; AuNPs, gold nanoparticles; Thi, thionine; PAT, phosphinothricin acetyltransferase.

## 5 Conclusion

In this study, a simple label-free electrochemical immunosensor for the ultrasensitive detection of PAT protein in GM crops was successfully constructed using AuNPs and Thi as signal amplification molecules. Using the label-free strategy, PAT protein could be detected based on the current changes caused by the immunoreaction on the electrode surface. Under optimal conditions, the limits of detection for soybean A2704-12 and maize BT-176 were as low as 0.02% and 0.03%, respectively. Following storage at 4°C for 33 days, the sensor still maintained 82.5% of the initial signal, with an RSD of 0.92%, exhibiting high selectivity, acceptable stability, high sensitivity, and good reproducibility. This method can provide an effective tool for the trace detection of GM crops. Moreover, research is ongoing for more targets to realize high-throughput analysis.

## Data Availability

The original contributions presented in the study are included in the article/Supplementary Material. Further inquiries can be directed to the corresponding authors.
