# Supplementary material for: A Label-Free Immunosensor Based on Gold Nanoparticles/Thionine for Sensitive Detection of PAT Protein in Genetically Modified Crops
Source: Front Chem. 2021 Dec 7;9:770584. doi: 10.3389/fchem.2021.770584 (PMC8688707; doi:10.3389/fchem.2021.770584)
Supplement: Supplementary file 1 [file Table1.DOCX]

Table S1 Seed powder standards of GM crops used in this study

| No. | Varieties | GM type | Content |
| --- | --- | --- | --- |
| 1 | Maize BT-176 | BT-Cry1Ac/PAT | 5% |
| 2 | Maize MIR604 | BT-Cry3A | 5% |
| 3 | Maize MON89034 | BT-Cry1A105/Cry2Ab | 5% |
| 4 | Maize MON88017 | CP4-EPSPS/Cry3Bb1 | 100% |
| 5 | Soybean RRS | CP4-EPSPS | 100% |
| 6 | Cotton MON88913 | CP4-EPSPS | 100% |
| 7 | Sugar beet H7-1 | CP4-EPSPS | 100% |
| 8 | Maize BT-11 | BT-Cry1Ab/PAT | 1% |
| 9 | Soybean A2704-12 | PAT | 1% |
| 10 | Rapeseed T45 | PAT | 1% |
| 11 | Maize MON810 | BT-Cry1Ab | 1% |
